# Value of Chromosome 9p21 Polymorphism for Prediction of Cardiovascular Mortality in Han Chinese Without Coronary Lesions: An Observational Study

**DOI:** 10.1097/MD.0000000000001538

**Published:** 2015-10-02

**Authors:** I-Te Lee, Kae-Woei Liang, Jun-Sing Wang, Wen-Jane Lee, Yii-der Ida Chen, Shih-Yi Lin, Wen-Lieng Lee, Wayne H.-H. Sheu

**Affiliations:** From the Division of Endocrinology and Metabolism, Department of Internal Medicine, Taichung Veterans General Hospital, Taichung (ITL, JSW, SYL, WHHS); School of Medicine, National Yang-Ming University, Taipei (ITL, KWL, SYL, WLL, WHHS); School of Medicine, Chung Shan Medical University (ITL, WHHS); Cardiovascular Center, Taichung Veterans General Hospital (KWL, WLL); Department of Medical Research, Taichung Veterans General Hospital, Taichung, Taiwan (WJL); and Institute for Translational Genomics and Population Sciences, Harbor–UCLA Medical Center, Torrance, California, USA (YDIC).

## Abstract

Variants at chromosome 9p21 are associated with coronary artery disease (CAD). However, the longitudinal effects of 9p21 variants on cardiovascular mortality remain controversial and may depend on whether the patient has CAD. We tested the hypothesis that the single-nucleotide polymorphism (SNP) rs4977574 is associated longitudinally with cardiovascular death in patients without detectable coronary lesions.

We enrolled patients who underwent coronary angiography for angina pectoris but had normal angiographic findings. Laboratory analyses and rs4977574 TaqMan genotyping were performed using fasting blood samples collected during hospitalization. Cardiovascular and all-cause mortality rates were acquired from a national database.

Among the 679 enrolled subjects with neither myocardial infarction nor an angiographic coronary lesion, 28 (19.0%) of the 147 homozygous GG carriers suffered a cardiovascular death, compared with 63 (11.8%) of the 532 subjects with the AG or AA genotype during the median 12.3 years (interquartile range 8.6–12.7 years) of follow-up. In a recessive model, cardiovascular mortality was significantly higher in subjects with the GG genotype than in those with the other genotypes (hazard ratio, 1.69, 95% confidence interval 1.08 to 2.64; *P* = 0.021).

In this follow-up study, rs4977574, a tag SNP at chromosome 9p21, was shown to be associated with cardiovascular mortality in Taiwanese patients with angina pectoris but no coronary lesions.

## INTRODUCTION

Genome-wide association studies have identified an association between chromosome region 9p21 and coronary artery disease (CAD). Several single nucleotide polymorphisms (SNPs) were initially identified in Caucasians ^1,2^ and the findings have been replicated in the Chinese population.^3–5^ In addition to CAD, variants at 9p21 are associated with stroke and peripheral artery disease.^6–8^ It is, therefore, possible that these SNPs could provide valuable information on the pathophysiology of cardiovascular disease (CVD), which is currently the leading cause of death worldwide.^9,10^

However, whether 9p21 variants can predict cardiovascular mortality has not been fully investigated. In subjects with established CAD, it has been reported that 9p21 variants were not associated with recurrent myocardial infarction or death,^11,12^ but an association between 9p21 variants and CAD death was observed in subjects with similar baseline CAD risks in the Global Registry of Acute Coronary Events.^13^ It was also recently reported that rs4977574, a tag SNP at chromosome 9p21, was significantly associated with new-onset cardiovascular events in patients without CAD.^14^ Therefore, to assess the longitudinal effects of 9p21 variants on CVD, it is important that the subjects have similar baseline coronary conditions.

The SNP rs4977574 was initially demonstrated to contribute to myocardial infarction risk in the Myocardial Infarction Genetics Consortium study.^15^ An association between rs4977574 and CAD has also been shown in several populations.^16–19^ However, it remains unknown whether rs4977574 can predict cardiovascular death in Chinese patients without CAD. We therefore assessed the rs4977574 polymorphism in a Taiwanese cohort of Han Chinese descent to test the hypothesis that this risk allele can predict cardiovascular death in Chinese patients without coronary lesions documented by angiography.

## METHODS

### Patients

Subjects undergoing coronary angiography due to angina pectoris were enrolled during hospitalization in the Cardiovascular Department of Taichung Veterans General Hospital between March 1999 and December 2000. Angiography was clinically indicated for the symptoms of chest pain with signs of ischemic heart disease based on the results of noninvasive assessments, including positive findings of exercise electrocardiography, nuclear imaging, or regional hypokinesia of the ventricular wall in the echocardiography. However, at the baseline, subjects with a history of myocardial infarction or coronary angioplasty were excluded. Patients were also excluded if the chest pain could be explained by other causes based on the inpatient record. Fasting blood samples were collected after an overnight fast. Clinical biochemical analyses were performed in the central laboratory during hospitalization, and only the residual buffy-coat component was stored at –80°C for all patients willing to donate their blood samples for academic use. Patients with a history of myocardial infarction or coronary revascularization were not included for analysis in this study. Patients were also excluded if luminal narrowing was detectable in any coronary artery based on the findings of coronary angiography. Mortality data were collected from enrollment. The buffy coat for genotyping had been delinked from personal identifiers and was analyzed anonymously after the clinical and mortality data had been collected based on the approval of the Institutional Review Board. The study protocol conforms to the ethical guidelines of the 1975 Declaration of Helsinki as reflected in a priori approval by the Institutional Review Board of Taichung Veterans General Hospital.

### Experimental Methods

Glucose levels were measured by the glucose oxidase–peroxidase method (Wako Diagnostics, Tokyo, Japan). Serum levels of cholesterol and triglyceride were measured by an enzymatic method using commercially available kits (Wako Diagnostics). Serum low-density lipoprotein (LDL) cholesterol was calculated for subjects with serum triglyceride levels below 400 mg/dL according to the method of Friedewald et al;^20^ otherwise, serum LDL cholesterol was measured after separation of very low-density lipoprotein from serum by ultracentrifugation and precipitation of apolipoprotein B (apo-B) containing particles with phosphotungstic acid and magnesium chloride reagent. Serum high-density lipoprotein cholesterol was measured after the precipitation of apo-B containing lipoproteins by phosphotungstic acid and magnesium chloride reagent.

Subjects’ genomic DNA was extracted from peripheral leucocytes using a QIAamp DNA Blood Mini Kit (Qiagen, Hilden, Germany). The SNP rs4977574 was genotyped using C_1754681_10 Assays-on-Demand SNP genotyping products (Applied Biosystems, Foster City, CA). The TaqMan genotyping reaction was amplified on a StepOnePlus Real-Time PCR System (Applied Biosystems) (95°C for 10 min, then 92°C for 15 s, and 60°C for 1 min for 40 cycles) and fluorescence was detected on the same instrument. Laboratory personnel were blinded to clinical phenotypes.

Coronary lesions were assessed on an angiography viewing workstation using software for quantitative analysis (Philips Inturis Suite, R2.2; Philips Medical Systems, Eindhoven, The Netherlands). All angiographic images were reviewed by the same cardiologist at major segments including: the left main, left anterior descending, left circumflex, and right coronary arteries; the first to third diagonal branches; the first to third obtuse marginal branches; and the posterior descending and intermediate arteries. Mortality data were collected until the end of 2011 from the Collaboration Center of Health Information Application, Department of Health, Executive Yuan, Taiwan. Causes of death were defined based on the diagnostic criteria of the International Classification of Disease, 9th Revision, Clinical Modification. Cardiovascular disease was defined as CAD (402, 404, 410, 411, 412, 413, 414, 426,427,428, 429), cerebrovascular disease (430, 431, 432, 433, 434, 435, 436, 437, 785.9), or peripheral artery disease (440.2, 443.8, 443.9, 444.2).^21^

### Statistical Analysis

All descriptive data are presented as the mean ± standard deviation. Categorical variables were analyzed using the *χ*^2^ test. The Hardy–Weinberg equilibrium was applied to test genotype distribution. The risk allele at chromosome 9p21 carried an odds ratio of 1.3 for having CVD.^22^ Therefore, a sample size of 404 subjects was required to detect differences with a 2-sided significance level of 5% and a statistical power of 80% based on an estimation using the Genetic Power Calculator.^23^ We employed one-way analysis of variance to assess continuous variables among groups. Cox proportional hazards regression analyses were used to determine cardiovascular and total mortality according to genotype. All statistical analyses were performed using SPSS 19.0 (IBM, Armonk, NY).

## RESULTS

**FIGURE 1:**
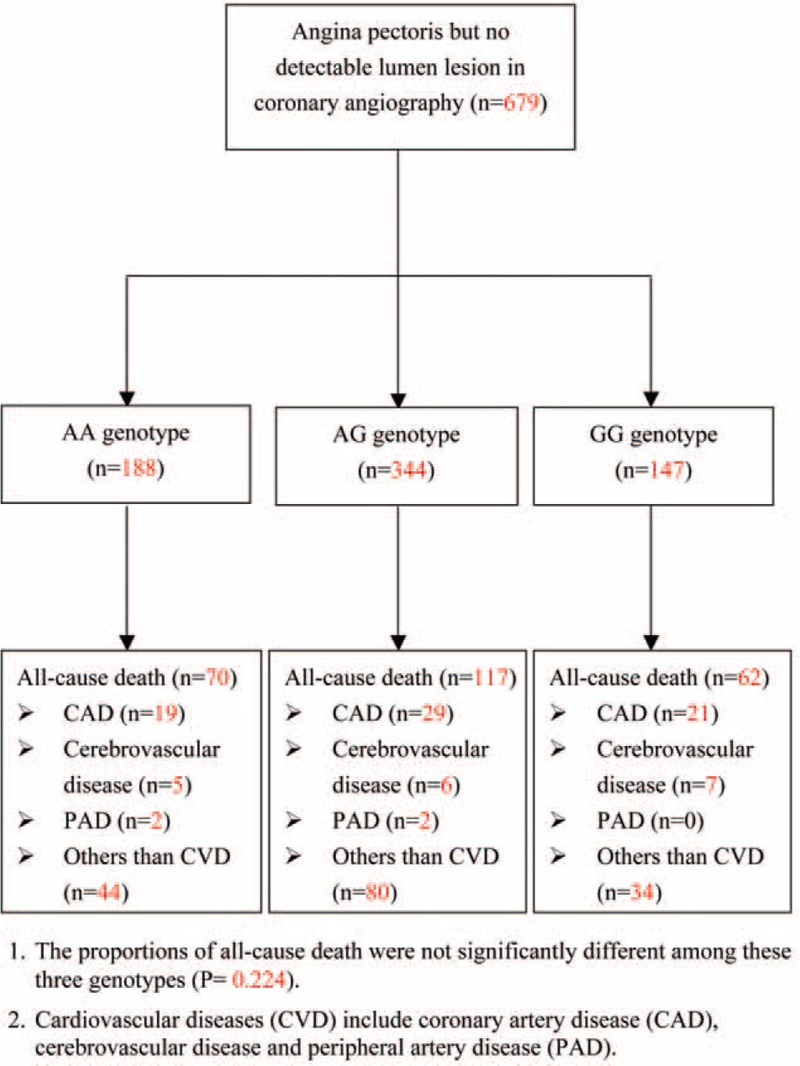
Enrollment of study subjects.

Among the 679 enrolled subjects with normal coronary angiography findings, genotyping of rs4977574 revealed that 188 had the AA genotype, 344 had the AG genotype, and 147 had the GG genotype (Figure 1). This genotype distribution was in agreement with the Hardy–Weinberg equilibrium (*P* = 0.658). There were no significant differences in age, sex, blood pressure, serum concentration of cholesterol or triglycerides, or fasting glucose (all *P* > 0.05) among the 3 genotype groups (Table 1).

**TABLE 1:**
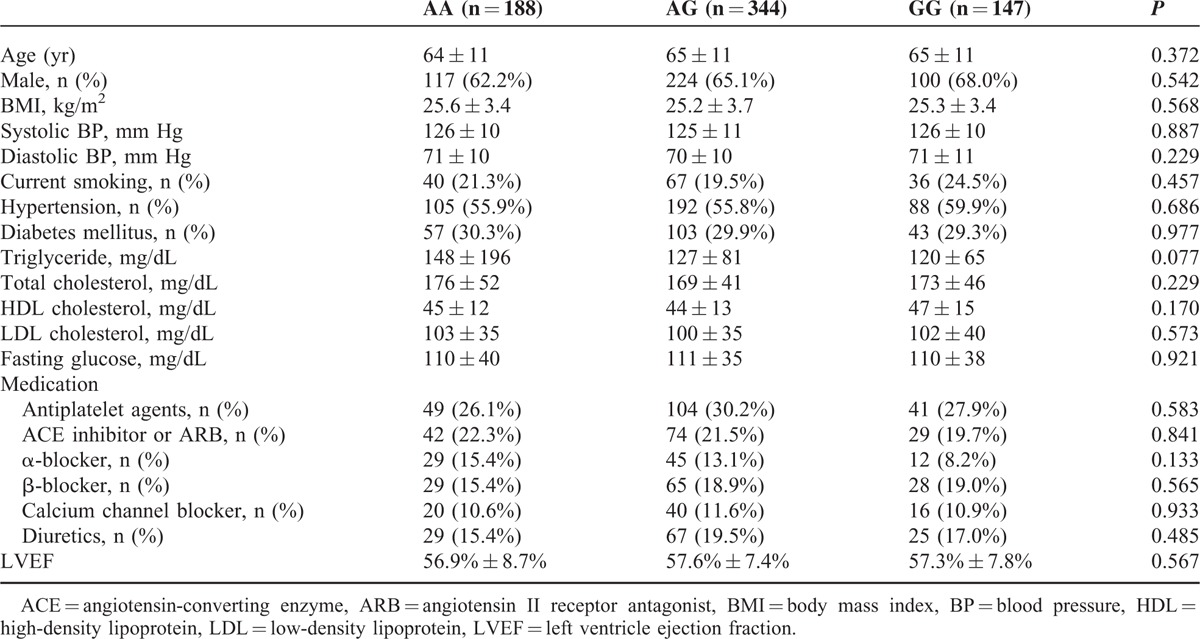
Baseline Characteristics of Subjects With Normal Angiography Findings

**FIGURE 2:**
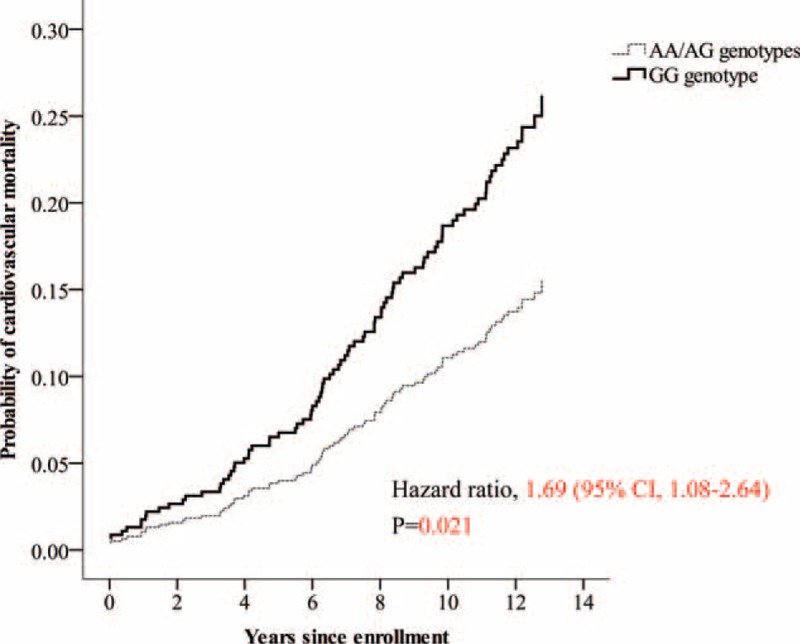
Cardiovascular mortality according to genotypes in subjects with normal coronary angiography findings at baseline.

**FIGURE 3:**
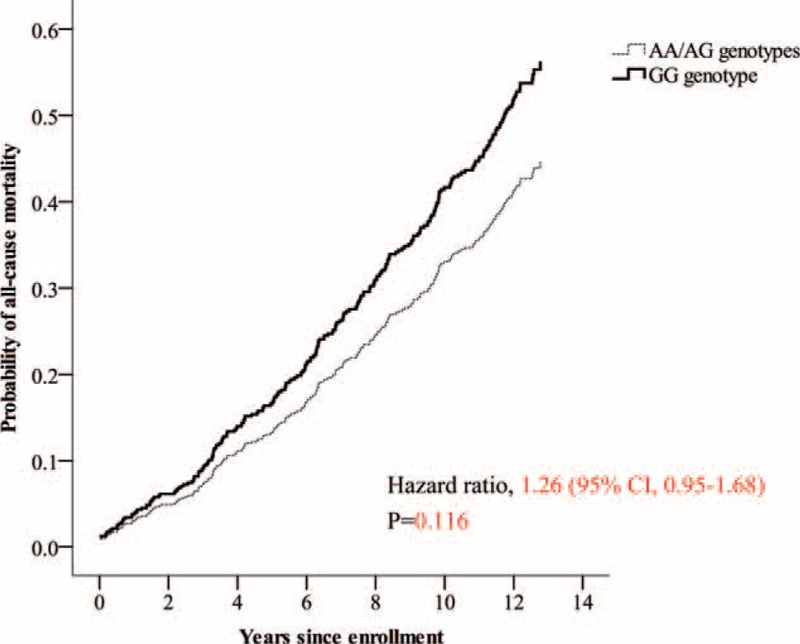
Total mortality according to genotypes in subjects with normal coronary angiography findings at baseline.

The median follow-up period was 12.3 years (interquartile range 8.6–12.7 yr). Twenty-eight (19.0%) of the 147 subjects with the GG genotype died due to CVD, compared with 63 (11.8%) of the 532 subjects with the AG or AA genotype. The risk of cardiovascular death was significantly higher (hazard ratio, 1.69; 95% confidence interval [CI], 1.08–2.64) in subjects with the homozygous GG genotype compared with the other subjects (Figure 2), but the total mortality risk was not significantly different (hazard ratio, 1.26; 95% CI, 0.95–1.68) (Figure 3). In the recessive model, the G allele predicted the risk of cardiovascular death with a hazard ratio of 1.82 (95% CI, 1.16–2.86; *P* = 0.009) and the risk of total mortality with a hazard ratio of 1.34 (95% CI, 1.00–1.79; *P* = 0.049) after adjusting for age, sex, smoking, hypertension, diabetes, and LDL cholesterol (Table 2).

**TABLE 2:**
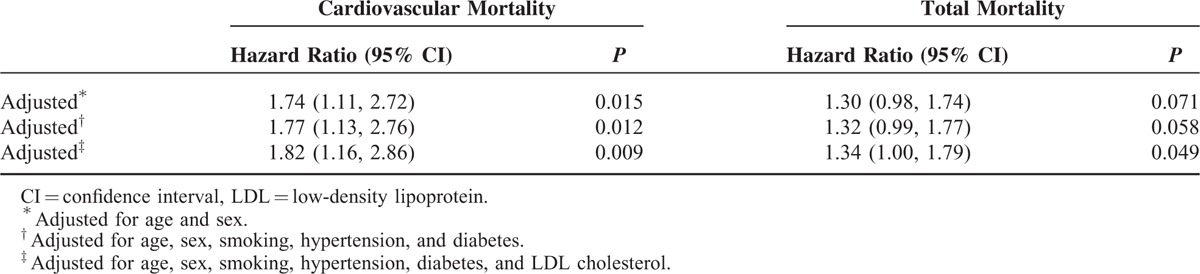
Effects of GG Genotype on Cardiovascular and Total Mortality in a Recessive Model on Cox Regression Analysis

## DISCUSSION

In the present study, we found that the rs4977574 polymorphism was significantly associated with cardiovascular mortality in Taiwanese subjects without coronary lesions. The symptom of anginal chest pain in subjects with normal coronary angiography findings is broadly described as cardiac syndrome X (CSX).^24,25^ In clinical practice, CSX does not appear to be rare in patients with chest pain.^26^ A variety of prognoses have been reported in the subjects with CSX. Kaski et al reported that none of the 99 CSX patients with a mean age of 45 years died in 7 years of follow-up.^27^ In the Women's Ischemia Syndrome Evaluation (WISE) study, the 5-year mortality rate was 3% in those women with normal coronary angiography at a mean age of 53.6 years.^28^ In the Coronary Artery Surgery Study (CASS), the 7-year mortality rate was 4% in subjects with a normal coronary angiography at a mean age of 49.6 years and 35% of mortality was caused by CVD. However, age was an important predictive factor, and the relative risk increased by approximately 18% for each additional year of age. In the present study, the subjects were older by a mean of 15 years than those in the CASS study.^29^ Generally, CSX has a relatively better prognosis than CAD in terms of mortality, according to previous reports,^30,31^ but the mortality rates in CSX subjects were still higher than those of a normal Chinese population.^32^ In the present study, the total mortality within the follow-up period was 36.7% in all subjects with CSX, representing 36.5% of the total mortality resulting from cardiovascular causes. Nonetheless, the total mortality was lower than that in subjects with established CAD, which was reported to be 37% in a 5-year cohort study by Cavender et al and 39% in an 11-year Vienna cohort study.^33,34^ In addition, cardiovascular mortality as a proportion of total mortality in subjects with CSX in the present study was much lower than that in CAD subjects (57%) in the Vienna cohort study.^34^ Although endothelial dysfunction has been suggested to be one of the mechanisms involved in CSX,^31^ prediction of mortality in subjects with CSX remains challenging as a result of various pathophysiological factors.^30,35,36^

The linkage disequilibrium block that contains SNP rs4977574 harbors a gene that encodes a large antisense noncoding RNA (*ANRIL*), which is expressed in endothelial cells.^37,38^ Expression of *ANRIL* is associated with atherosclerosis and may be involved in the regulation of the cyclin-dependent kinase inhibitor 2A and 2B genes.^39,40^ The *MTAP* gene, which encodes methylthioadenosine phosphorylase, an essential enzyme in polyamine metabolism, has also been found to be associated with atherosclerosis. Furthermore, over-expression of *ANRIL* is also associated with increased cell proliferation, increased cell adhesion, and decreased apoptosis.^41^ The potential mechanism that links between 9p21 variants and CVD death remains unclear. However, investigation of chromosome 9p21 variants may provide a better understanding of the mechanisms of CVD in patients with CSX.^42^

It has been well documented in cross-sectional studies that chromosome 9p21 variants are associated with CAD.^43^ However, several prospective studies have suggested that 9p21 variants cannot predict CVD in subjects with established CAD.^11,12,44^ Null hypothesis findings might result from variations in the severity of, or interventions for, coronary lesions at the baseline.^11,45^ It has been reported that 9p21 variants were not associated with recurrent coronary events in CAD patients treated with drug-eluting stents.^45^ In contrast, it has been reported that rs4977574 could predict cardiovascular events with a population attribution risk of 13% (reported *P* value less than 0.001) in subjects without CAD during a median follow-up period of 11.7 years;^14^ this significant finding in Caucasians is mirrored by our works in the Chinese population with a similar follow-up period. The present study investigated an older population (mean age of 64.3 vs. 58.0 yr) with angina pectoris, whereas the study by Gränsbo, et al^14^ analyzed a normal population. However, one of the main strengths of our study was that the findings were derived from subjects without detectable coronary lesions proven by angiography at the baseline. The impact of chromosome 9p21 variants in CAD is similar between Asians and Caucasians.^22^ Our findings provide longitudinal evidence that chromosome 9p21 variant could predict CVD mortality in Chinese subjects with CSX. Therefore, we recommend that CVD prevention strategies should classify patients with the risk rs4977574 allele as a population with a higher CVD risk than those without the risk allele. Further studies for CVD prevention are thus warranted.

There were limitations in the present study. First, the mortality data were obtained from a National Health Insurance registry. Although this provides a nationwide coverage rate of over 99% in Taiwan,^46^ nonfatal cardiovascular events cannot be traced using this database. Second, our data showed a trend toward higher rate of mortality in subjects with the GG genotype than in those with the GA and AA genotypes, but the hazard ratio was not as high as for CVD mortality, and only reached the statistical significance after adjusting for age, sex, smoking, hypertension, diabetes, and LDL cholesterol. A weak association with total mortality might result from similar rates of the mortality other than CVD (23.1% in GG genotype vs. 23.3% in AG or AA genotypes, *P* = 0.999). Furthermore, we were unable to address the influence of the environment or treatment effects during the follow-up period.

In conclusion, the chromosome 9p21 polymorphism rs4977574 could predict cardiovascular mortality in Taiwanese Han Chinese individuals with angina pectoris but without any history of CAD or detectable coronary lesions, in study with a 12.3-year follow-up period.
